# Mass Potentials Recorded at the Round Window Enable the Detection of Low Spontaneous Rate Fibers in Gerbil Auditory Nerve

**DOI:** 10.1371/journal.pone.0169890

**Published:** 2017-01-13

**Authors:** Charlène Batrel, Antoine Huet, Florian Hasselmann, Jing Wang, Gilles Desmadryl, Régis Nouvian, Jean-Luc Puel, Jérôme Bourien

**Affiliations:** 1 INSERM—UMR 1051, Institute for Neurosciences of Montpellier, Montpellier, France; 2 University Montpellier, Montpellier, France; Universidad de Salamanca, SPAIN

## Abstract

Auditory nerve fibers (ANFs) transmit acoustic information from the sensory hair cells to the cochlear nuclei. In experimental and clinical audiology, probing the whole ANF population remains a difficult task, as the ANFs differ greatly in their threshold and onset response to sound. Thus, low spontaneous rate (SR) fibers, which have rather higher thresholds, delay and larger jitter in their first spike latency are not detectable in the far-field compound action potential of the auditory nerve. Here, we developed a new protocol of acoustic stimulation together with electrophysiological signal processing to track the steady state activity of ANFs. Mass potentials at the round window were recorded in response to repetitive 300-ms bursts of 1/3 octave band noise centered on a frequency probe. Analysis was assessed during the last 200-ms of the response to capture the steady-state response of ANFs. To eliminate the microphonic component reflecting the sensory cells activity, repetitive pairs of sounds of opposite polarities were used. The spectral analysis was calculated on the average of two consecutive responses, and the neural gain was calculated by dividing point-by-point the spectrum to sound over unstimulated condition. In response to low-sound-level stimulation, neural gain predominated in the low-frequency cochlear regions, while a second component of responses centered on higher cochlear frequency regions appeared beyond 30 dB SPL. At 60 dB SPL, neural gain showed a bimodal shape, with a notch near 5.6 kHz. In addition to correlate with the functional mapping of ANFs along the tonotopic axis, the deletion of low-SR fibers leads to a reduction in the high-frequency response, where the low-SR fibers are preferentially located. Thus, mass potentials at the round window may provide a useful tool to probe the SR-based distribution of ANFs in humans and in other species in which direct single-unit recordings are difficult to achieve or not feasible.

## Introduction

Within the cochlea, the mechano-transduction is achieved by sensory inner hair cells (IHCs) that convert acoustic stimulus into trains of action potentials along afferent auditory nerve fibers (ANFs). The ANFs show a great diversity in term of threshold, characteristic frequency (CF), and spontaneous rate (SR) of discharge, to encode sounds over a large intensity and frequency range [1,2]. For a given fiber, the SR is negatively correlated with threshold [1], making the high-SR fibers more sensitive to lower sound pressure levels whereas low-SR fibers are rather recruited at higher sound pressure levels.

The earliest single-unit studies of ANFs reported firing synchronization to the fine structure of tones, in the sense that discharges occur at a preferential timing of the cyclical waveform [3,4,5,6,7]. For example, ANFs have the striking capability to “phase-lock” to low-frequency tones up to several kHz [8]. Above this frequency limit of neural phase-locking, inner hair cell (IHC) membrane potential cannot follow the sound stimulation waveform [9] and frequency coding relies on the place where each frequency produces vibrations along the basilar membrane (i.e., cochlear tonotopy). Phase-locking also occurs to stimulus envelope ([10] for a review). Both forms of phase-locking are readily apparent in the peri-stimulus time histogram to the amplitude modulated stimulus [11,12,13]. The range of modulation frequencies encoded by a single ANF is well characterized by a low-pass modulation transfer function with a 3-dB cut-off frequency, which increases with CF of the fibers [11].

The sound-evoked compound action potential (CAP) and its corresponding wave I of the auditory brainstem responses (ABR), which reflect the synchronous activity of ANFs at the stimulus onset, are commonly used to probe deafness in both experimental and clinical settings. However, based on paired recordings of single units and sound-evoked CAP, we reported previously that the delay of the first-spike latency and its large jitter make low-SR fibers unlikely to contribute to triggered CAP [14]. Thus, CAP and ABR wave 1 are not satisfactory measures for tracking all the ANF pools. Alternative detection tools are thus particularly important as i) low-SR fibers insure the coding of a large dynamic range of sound pressure levels [2], ii) low-SR fibers are finely phase-locked to the stimulus waveform envelope [11,12,13], which is essential for speech intelligibility, and iii) low-SR fibers are of a primary importance for signal detection in noisy environments [2,15].

Here, we develop a new protocol of acoustic stimulation and electrophysiolocal signal processing to detect the activity of ANFs, including the low-SR fiber pool. To do this, we analyzed mass potentials from an electrode placed on the round-window niche [16,17]. In absence of sound stimulation, this activity is known to reflect the asynchronous activity of the ANFs assembly [18,19,20] which is dominated by the high-SR fiber pool. The hallmark of this electrical neural noise corresponds to a spectral component that is best described by a power spectrum density (PSD) with a predominant peak near 900 Hz [18]. In response to sound stimulation, the mass potential contains both a neural component coming from ANFs, and a microphonic component originating from transduction currents in hair cells [21]. To eliminate the microphonic component, we used repetitive pairs of sounds of opposite polarities, and the neural gain was calculated by dividing point-by-point the spectrum to sound over unstimulated condition. Here, we showed that i) the amplitude of the neural gain correlates with the functional mapping of ANFs along the tonotopic axis [2,14,22,23,24], and ii) the deletion of low-SR fibers leads to a reduction in the high-frequency response (where the low-SR fibers are preferentially located), while the amplitude of CAP of the auditory nerve does not change.

## Materials and Methods

### 1. Ethics statement

Female Mongolian gerbils (50–61 g, 8 to 12 weeks of age) were obtained from Janvier Labs (Saint-Isle, France). Animals were housed in facilities accredited by the French “Ministère de l’Agriculture et de la Forêt” (Agreement C-34-172-36; December 19, 2014). Animals facilities were maintained at 24°C, 60–80% relative humidity, on a 12 h light–dark cycle and the animals were allowed free access to food and water. Experiments were carried out in accordance with the animal welfare guidelines 2010/63/EC of the European Communities Council Directive regarding the care and use of animals for experimental procedures. Animals were housed in facilities accredited by the French “Ministère de l’Agriculture et de la Forêt” (Agreement C-34-172-36), and the experimental protocol was approved (Authorization CEEA-LR-12111) by the Animal Ethics Committee of Languedoc-Roussillon (CEEA-LR36; France). At the end of functional examination, gerbils were killed by cervical dislocation under deep anesthesia (Pentobarbital 50 mg/kg). A total number of 30 animals were used, of which 23 animals provided useful data as follows: 9 were infused with artificial perilymph into the round window niche during 30 minutes (among of them 5 were used for immunocytochemistry); 10 was infused with artificial periphymph containing 33 μM ouabain (among of them 5 were used for immunocytochemistry), 4 infused with artificial periphymph containing 10 μM TTX. All efforts were made to minimize the number and suffering of the animals used.

### 2. Drug preparation

Artificial perilymph solution consisted of the following (in mM): 137 NaCl; 5 KCl; 2 CaCl2; 1 MgCl2; 1 NaHCO3; 11 glucose; pH 7.4; osmolarity: 304 ± 4.3 mOsm/kg. Before each experiment, ouabain (Sigma, St. Louis, MO, USA) and tetrodotoxin (TTX, Latoxan, Portes les Valence, France) were prepared in artificial perilymph to a final concentration of 33 μM and 10 μM, respectively.

### 3. Surgery and round window infusion technique

Electrophysiological recordings were performed in anesthetized gerbils via a plug fixed on the skull and linked to the round window electrode. Gerbils were anesthetized by an intraperitoneal injection of a mixture of 3 mg/kg Xylazine (Rompun®2%) and 40 mg/kg tiletamine/zolazepam (Zoletil®50). Electrocardiogram (EKG) and withdrawal reflex (lack of response to toe pinch) were used to insure a deep anesthesia and evaluate the physiological status of the animals. Supplemental doses of Xylazine and tiletamine/zolazepam were administered as needed.

The left cochlea was exposed through a dorsal approach. Once the bulla had been opened, the recording electrode was placed on the bony edge of the round window membrane, leaving enough space in the round window niche for the infusion glass pipette. The infusion glass pipette was filled with artificial perilymph alone or containing ouabain and was introduced into the round window niche (leaving the round window intact) using a micromanipulator (Warner Instruments). The infusion pipette was connected to a syringe pump (Warner Instruments), which pushed out the solution at a rate of 150 μl/h. After 30-min infusion, the solutions were wicked away from the round window niche, and the bulla (including the recording electrode) was closed with dental cement. The round window and the reference electrode placed in the neck were soldered to a plug fixed on the skull. The surgical site was then cleared and the skin wiped with a topical antiseptic (Betadine). In the days immediately after surgery, animals were weighed for evidence of weight stability/gain, and observed for evidence of good mobility in their cage.

### 4. Electrophysiological recordings

Six days after artificial perilymph (control) or 33 μM ouabain perfusion, electrophysiological recordings were performed under anaesthesia (3 mg/kg Xylazine and 40 mg/kg tiletamine/zolazepam) in a Faraday shielded anechoic sound proof cage. The animal’s rectal temperature was measured with a thermistor probe and maintained at 38°C ± 1°C using a heating blanket. The acoustical stimuli were delivered under calibrated conditions using a custom acoustic assembly set-up including a signal generator (PXI-4461 controlled by LabVIEW, National Instrument Company), an audio amplifier (Tucker Davis, SA1) and a magnetic speaker (Tucker Davis, MF1).

The compound action potential (CAP) was recorded from the round window electrode in response to 10-ms tone bursts at 2 to 32 kHz (1-ms rise/fall, 11 bursts/s, alternating polarity). Amplification of the cochlear signal (×20,000) was achieved by a Grass P511 differential amplifier with a 100 Hz to 3.5 kHz bandpass. CAP amplitude was measured between N_1_ and P_1_, the threshold being defined as the dB SPL needed to elicit a measurable response (> 2 μV).

**Fig 1:**
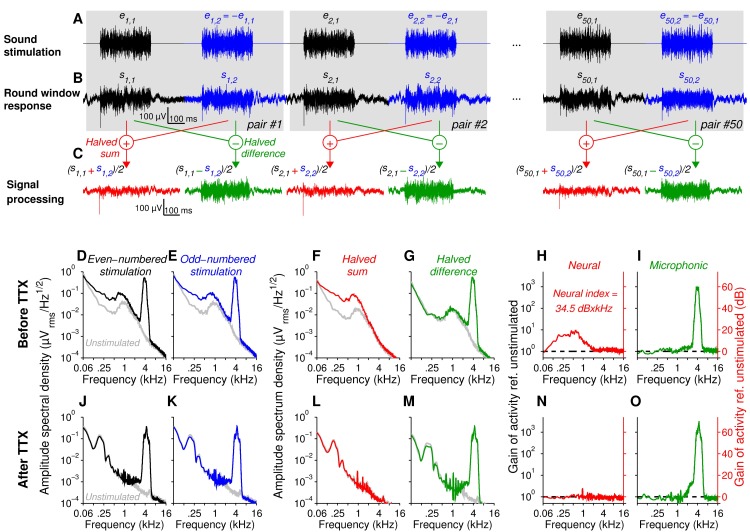
Extraction of a neural index from the round-window response. **(A, B)** Round-window responses (bottom, *s*) were evoked by repetitive 300-ms bursts of third-octave band noise (top, *e*) centered at a probe frequency (50 dB SPL and 4 kHz in this example). To reduce the cochlear microphonic: *i*) two consecutive stimuli (that form a pair) were presented in opposite polarity and *ii*) all the pairs (*n* = 50) differed from each other (by changing the seed of the pseudorandom stream). (**C**) Calculation of halved sum (red) and halved difference (green) within each pair of response using black and blue traces shown in **B**. (**D-O)** Spectral analysis of round-window response before (**D-I**) and after 10 μM TTX application (**J-O**) into the round window niche. The grey trace is the power spectral density of the signal recorded at the round window in the absence of sound stimulation (unstimulated activity). Black and blue traces (**D, E**) are calculated from responses with even-numbered (black, first element of each pair) or odd-numbered rank (blue, second element of each pair). Note the mixture of a neural component centered around 900 Hz and the microphonic centered at 4 kHz. To segregate neural and microphonic components, the spectral analysis was calculated after the half summation (red, **F**) or the half difference (green, **G**) of traces within each pair of response (see equations in inset). The neural and microphonic sound-evoked activities (red and green traces in **H** and **I**) were derived from the traces shown in **F** and **G** respectively (ratio between colored and grey traces). Note the complete disappearance of neural activity after TTX application, leaving the cochlear microphonic unaffected (**J-O**). The area under the red curve in panel **H** was used as an index of neural sound-evoked activity (34.5 dB×kHz in this example).

The power spectral density (PSD) of electrical neural noise was recorded in the absence (unstimulated) or in response to sound stimulation (stimulated) using the same electrode-amplifier assembly dedicated for CAP measurement but with a 1 Hz to 30 kHz bandpass. Acoustic stimuli consisted of bursts of one third–octave band noise centered at a probe frequency varying from 1 to 32 kHz in 1/3 octave steps (2.5-ms rise and fall, 300-ms on/300-ms off, 100 presentations per level). A single PSD estimate using Welch’s method ([25]; *pwelch* function using Matlab language, 2048 samples per segment, 50% overlapped, rectangular window, sampling rate 50,000 samples/sec) was calculated on each trial during the last 200-ms of the sound stimulation, and the mean PSD was then calculated by averaging all the PSDs. With this analysis time window, we excluded on-set [26,27] and off-set [28] adaptation responses of fibers. A detailed description of the method is provided in the Result part and **Fig 1**.

### 5. Contribution of unitary action potential at the round window

The unitary contribution (unit action potential) to the response at the round window was computed according Kiang and co-workers [29]. In absence of sound stimulation, each spontaneous action potential fired by one fiber in the auditory nerve is used as a trigger pulse to average its corresponding unitary action potential recorded at the round window[30]. The method to record single auditory nerve fibers in gerbils has been described elsewhere [2,14].

The contribution of the unit action potential to the electrical neural noise in response to amplitude modulated bandlimited noise was simulated by convolving the waveform of unitary contribution with a stereotyped modulation transfer function (lowpass Butterworth filter, 8th order) according to [11]. The 3-dB cut-off frequency of low-pass filters was automatically adjusted with a derivative-free method (*fminsearch* function using Matlab language) to match experimental data at 60 dB SPL.

### 6. Number of ribbon-synapses per inner hair cell along the tonotopic axis

The immunohistochemistry method to assess the number of synapses per inner hair cell (IHC) has been extensively described [14]. Briefly, the presynaptic IHC ribbons were identified using a mouse anti-CtBP2 antibody (1:500; BD Biosciences, San Diego, CA). Glutamate receptors were labelled with a mouse antibody raised against the C-terminus of the GluA2 subunit, IgG2a (1:200, Millipore, Billerica, MA). A 3D, custom algorithm was used to detect the juxtaposition of pre- and post-synaptic structures in stacked confocal images.

### 7. Data analysis

Means were expressed ± S.E.M. The significance of the group differences was assessed with a two-ways ANOVA; given significance of the group differences (*p* < 0.05), a Tukey's *post hoc* tests were subsequently used for pairwise comparisons; *p<0.05, and **p<0.01. Data analysis was performed using Matlab (The Mathworks Company) and its Statistics, Signal, and Image toolboxes.

## Results

In the absence of sound stimulation, the PSD of electrical round-window noise exhibited maximal energy at the lowest frequencies, reaching a minimum at ~ 400 Hz (422 Hz ± 19, mean ± SEM, *n* = 30) and a broad peak centered on ~ 900 Hz (915 Hz ± 16, mean ± SEM, *n* = 30, Fig 1). To probe the efficiency of PSD amplitude for the detection of the subtle loss of low-SR fibers, we compared sound-evoked responses recorded 6 days after a 30 min infusion of 33 μM ouabain into the round window niche (*n* = 9) to those of controls obtained with control artificial perilymph (*n* = 10).

### 1. Sound-driven neural activity at the round window

Mass potentials at the round window were recorded in response to 300-ms bursts of 1/3 octave band noise centered on a frequency probe (Fig 1A). The PSD of mass potentials was assessed during the last 200-ms of the response, to capture the steady-state response of ANFs. To extract the neural activity and cochlear microphonic, reflecting the auditory-nerve and sensory hair-cell activity, we used repetitive pairs of noise bursts of opposite polarities, all the pairs differing from each other in their temporal structure (Fig 1A and 1B). When estimated over the first or second element of the 50 pairs (black and blue traces in Fig 1A and 1B, respectively), the PSD is characterized by a mixture of both neural and microphonic components (Fig 1D and 1E). In each pair of bursts having opposite polarities, summed temporal response drastically reduces the microphonic potential, isolating the neural component (red traces in Fig 1C and its PSD in Fig 1F). In contrast, the difference between the temporal traces within each pair resolves the microphonic component only, *i*.*e*., the amplitude of the 900-Hz peak was comparable to the unstimulated activity (see green traces in Fig 1C and its PSD in Fig 1G).

The gain of activity (neural and microphonic) evoked by sound stimulation was measured by dividing point-by-point the PSD measured in response to sound by the PSD in unstimulated condition (Fig 1H and 1I). When this ratio is closed to 1 (*i*.*e*. 0 dB in gain), there is no gain of activity evoked by sound. Inversely, a ratio above 1 indicates a significant gain of activity that can be expressed in dB (see double axes in Fig 1H and 1I). Consistent with its neural origin, the acute 30-min round window infusion of 10 μM TTX (*n* = 4) abolished the neural response, leaving the cochlear microphonic unaffected (Fig 1J–1O). We sometimes observed a 200 Hz peak after TTX infusion, *i*.*e*., when the neural activity was inhibited. Although this component has been attributed to the activity of the dorsal cochlear nucleus [20] or to be a manifestation of tinnitus [31], the lack of effect in response to increasing sound stimulation of this 200 Hz component suggests an origin in extra-auditory structures (Fig 1J–1M).

**Fig 2:**
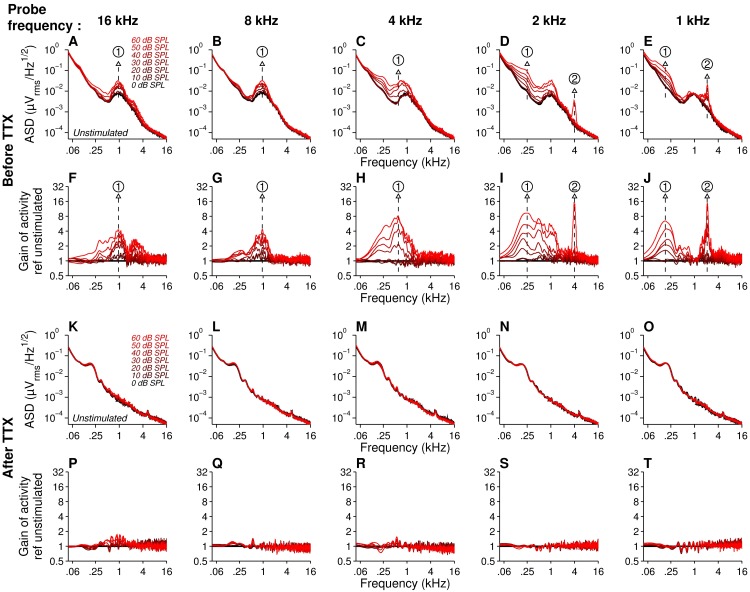
Neural subcomponents in round window responses. Spectral analysis of the halved sum responses across frequency (1 to 16 kHz) and intensity (0 to 60 dB SPL), before (1^st^ row, power spectral density, **A-E,** 2^nd^ row, gain of activity, F-**J**) and after 10 μM TTX application (1^st^ row, power spectral density, **K-O,** 2^nd^ row, gain of activity, **P-T**). PSD recorded in the absence of sound stimulation are shown in black (unstimulated activity). PSD obtained in response to level of stimulation from 0 to 60 dB SPL in 10 dB increments are shown in red. The spectral subcomponent (1), which reflects the steady-state firing of fibers, decreases in frequency for probe frequency below 8 kHz (C-E and H-J). The subcomponent (2) set at twice the stimulus frequency is a second harmonic resulting of half summation.

**Fig 3:**
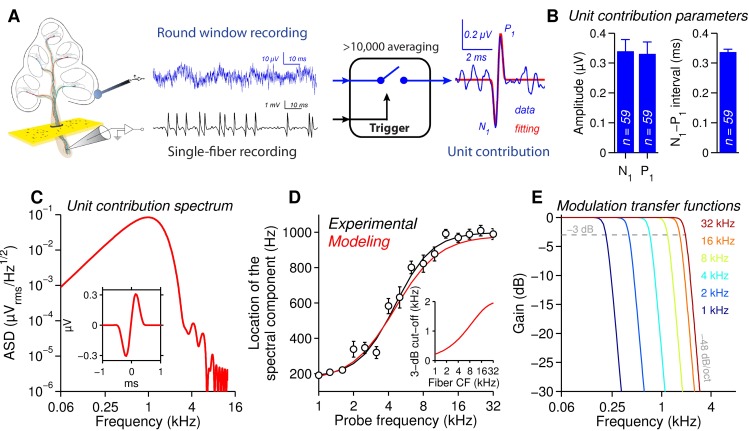
Low-pass modulation transfer functions account for spectrum shift behaviour. **A.** Protocol used to record unit contribution at the round window (from [29,30,33]). Spontaneous action potentials (black trace) recorded with an electrode in the auditory nerve were used as trigger pulses to average the corresponding action potentials recorded with a gross electrode at the round window (blue trace). After more than 10,000 averages, a biphasic waveform of 0.3 μV amplitude, 1 ms second duration, was obtained. Red fit: *f(t) = A×((cos(2πf*_*1*_*t)+1)×sin(2πf*_*2*_*t)* with *A* = 0.31 ± 0.04 μV, *f*_1_ = 997 ± 20 Hz, *f*_2_ = 920 ± 33 Hz, *R*^2^ = 0.95 ± 0.007, 59 fibers, >10,000 averaging per fiber. **B**. Parameters of the unit contribution (*n* = 59 ANFs). **C**. Amplitude of the spectrum density function (ASD) of the unit contribution (adequate zero padding was applied to improve the frequency resolution of the spectral estimate). The peak in the ASD is around 1 kHz. *Inset*: Unit contribution model used to estimate the PSD. **D**. Location of the spectral component as a function of the probe frequency for experimental (black curve, 10 gerbils) and simulated data (red curve) at 60 dB SPL. Note the spectrum shift for a probe frequency below 10 kHz. *Inset*: 3-dB cut-off frequency of modulation transfer functions as a function of the fiber CF (*f(CF) = 2000×(1-exp(-CF/9000))* with *CF* in Hz). **E**. Low-pass modulation transfer functions obtained from **C** and **D** (8^th^ order Butterworth filters, 0 dB in band pass and cut-off frequency at 3-dB) for fibers with CF ranging from 1 to 32 kHz in 1 octave steps. Note that cut-off frequency is positively correlated with the CF of the fibers.

We then quantify the neural gain across frequency and intensity. When the cochlea was stimulated at 16 kHz and 8 kHz, neural gain increased monotonically according to sound levels, with a predominant peak in the PSD curve around 900 Hz (Fig 2A and 2B and Fig 2F and 2G). For sound stimulation from 4 to 1 kHz, the maximum gain peak shifts to the left, *i*.*e*. toward the low-frequency spectral component from 600 to 200 Hz, respectively (582 ± 43 Hz at 4 kHz, 338 ± 45 Hz at 2 kHz, and 191 ± 3 Hz at 1 kHz, Fig 2C–2E and Fig 2H–2J). This frequency shift can be explained by occurrence of low frequency fluctuations in spike train in response to low-frequency envelope modulations in the stimulus, consistently with a low-pass modulation transfer function [10,11,32]. In theory, convolution of the “unitary action potential” of the auditory nerve recorded from the round window [29,30,33] with low-pass modulation transfer function should recapitulate the frequency shift we observed in the neural gain. To address the hypothesis, we first simulate the experimental waveform of unitary action potential recorded in 5 normal-hearing gerbils (*n* = 59 fibers, >10,000 averaging per fiber, CF ranging from 0.89 to 48 kHz, SR ranging from 3 to 130 spikes/sec, see inset in Fig 3A–3C). We then reproduce the shift of the peak toward low-frequency range of the spectrum (Fig 3D) by applying low pass filter transfer function (Fig 3E) with a 3-dB cut-off frequency positively correlated with CF (210 Hz at CF = 1 kHz, 400 Hz at CF = 2 kHz, 720 Hz at CF = 4 kHz, 1180 Hz at CF = 8 kHz, 1660 Hz at CF = 16 kHz, 1940 Hz at CF = 32 kHz, see Fig 3D inset).

In addition, an harmonic at twice the probe frequency appeared in neural gain in the summed response to 1 and 2 kHz third-octave band noise (Fig 2D, 2E, 2I and 2J). Due to the non-linear nature of the ANF response [16,17,34,35], the low-frequency neurophonic response consists of multiple harmonics (dominated by a strong 1^st^ and 2^nd^ harmonic). These harmonics were divided over the sum and difference responses (see Fig 1 and Fig 2). For the fine-structure-neurophonic, the summed response contains all even harmonics and is dominated by the second harmonic, whereas difference response contains all odd harmonics, dominated by the first. To control that the second harmonic measured in the summed response originates from neurophonic, and does not correspond to a microphonic component, we applied TTX into the round window niche and retested the animal using the same protocol (Fig 2K–2T). Indeed, the neurophonic responses including the second harmonic disappeared after TTX application.

### 2. Neural gain index reflect the SR distribution of ANFs

**Fig 4:**
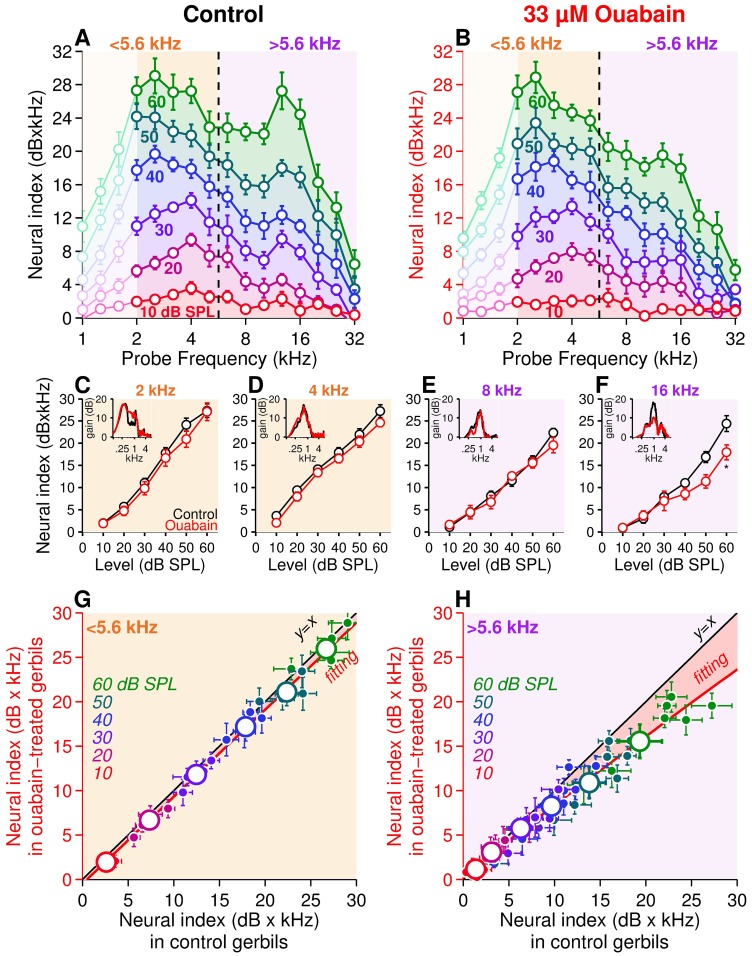
Probing the auditory nerve using round-window response in control and ouabain-poisoned cochleae. **(A, B)** Iso-level neural index in control (**A**, *n* = 10 gerbils) and ouabain-poisoned cochleae (**B**, *n* = 9 gerbils). The frequency probe varied from 1 to 32 kHz in 1/3 octave steps and sound level from 10 (red curve) to 60 dB SPL (green curve) in 10 dB steps. Note that below 2 kHz (light-coloured area), the neural index amplitude decreases because of the phase locked response of fibers and their cancelation by opposite polarities. (**C-F**) Amplitude *versus* intensity functions of the neural index at 2 (**C**), 4 (**D**), 8 (**E**), and 16 kHz (**F**), in artificial perilymph control (black) and ouabain-poisoned cochleae (red). *Inset*: examples of neural sound-evoked response in control (black) and ouabain-poisoned cochleae (red) as shown in Fig 1G. (**G, H**) Ouabain-poisoned *versus* control correlations from low- (< 5.6 kHz, **G**) and high-frequency probe (> 5.6 kHz, **H**) derived from panels A and B. The data obtained below 2 kHz were excluded. The coordinate of each small dot corresponds to the neural index amplitude in control (*x*-coordinate) and ouabain condition (*y*-coordinate) measured for the same frequency and sound level. Large dots represent the average of small dots pooled per sound level (from 10 dB SPL in red to 60 dB SPL in green). The black line is the invariant model *y* = *x*, simulating an absence of drug effect. Red curves are lowest-order polynomial fits to the data (G, *y* = 0.96×*x—*0.06, *r*^2^ = 0.96; H: *y* = -0.002×*x*^2^+0.8×*x*+0.18, *r*^2^ = 0.97). Data are expressed as the mean ± SEM. *X* and *Y* error bars display the mean ± SEM of data shown in *x* and *y* axis, respectively; * *p*<0.05, two-way ANOVA test followed by *post hoc* Tukey’s test.

The area under the curve of the neural gain (in dB) was then measured as a neural gain index (Fig 1H) and plotted as a function of the probe frequency (Fig 4A). The iso-level frequency response of the neural gain showed different spectral patterns according to the level of sound stimulation. At low stimulation levels (below 20 dB SPL), the neural gain index predominated in the 4 kHz frequency range. Up to 30 dB SPL, a second response component centered on the 12 kHz appeared. At 60 dB SPL, the iso-level function displayed a two-peak shape with a low frequency peak at 2.5 kHz and a high frequency peak at 12.5 kHz (Fig 4A). Although the number of synapses per IHC reached about 20–25 synapses per IHC (see [36]) in the 1–2 kHz region of the cochlea, a drastic reduction in the neural gain index was seen for probe frequencies below 2 kHz. This neural cancelation may be due to the phase-locking behavior of the ANFs to fine structure presented in alternating phase (Fig 2E and 2J, [16,34,35]). At these low-frequencies of stimulation, the fine-structure of the waveform follows the stimulus polarities, eliciting phase-locked firing of the ANFs in a 180° phase shift (i.e., 1/2 cycle shift). In far field recordings, the summation of the response for 2 stimuli of opposite polarities will cancel the phase-locked neural activity, and thus account for the poor sensitivity of the 900-Hz peak to sound stimulation (Fig 2E and 2J). To further test that the bimodal shape of the sound-driven neural responses reflected the SR-based distribution in the auditory nerve of gerbil, we therefore only consider neural gain index in response to acoustic stimulation centered on frequencies above 2 kHz for further analysis.

**Fig 5:**
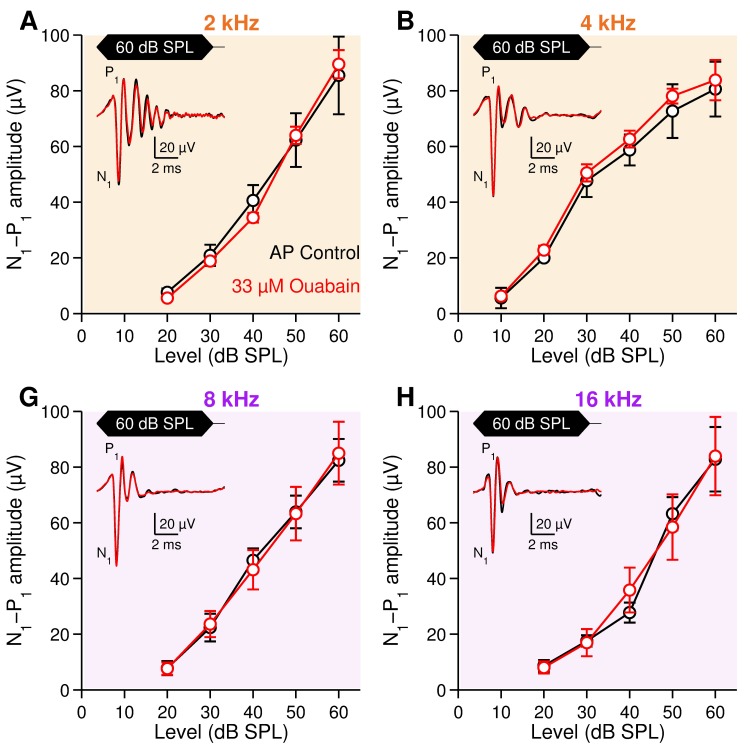
Compound action potential of the auditory nerve in control and ouabain-poisoned cochleae. CAP amplitude-intensity functions in response to 2 (**A**), 4 (**B**), 8 (**G**), and 16 kHz (**H**) tone bursts, in artificial perilymph control (black, *n* = 10) and ouabain-poisoned cochleae (red, *n* = 9). *Inset*: Example of CAP in control (black) and ouabain-poisoned cochleae (red). CAP amplitude was measured between N_1_ (the first negative wave) and P_1_ (the subsequent positive wave). Data are mean ± SEM. No statistical difference was found between control and ouabain-perfused animals. *p* > 0.05, two-way ANOVA.

Next, we probe the sensitivity of the neural index to the loss of low-SR fibers by infusing ouabain into the round window niche [14]. Six days after a 33 μM ouabain infusion, the neural index was significantly decreased in the 12 kHz frequency range, leaving the lower frequency region below 5.6 kHz, unaffected (Fig 4B–4F). We then compared the neural gain index after ouabain infusion with artificial perilymph at the same frequency and sound-stimulation level (Fig 4G and 4H). Below 5.6 kHz, the neural gain index showed a linear relationship between the poisoned and the control cochleae, indicating that ouabain has no effect on the neural activity in the low-frequency range (Fig 4G). In contrast, above 5.6 kHz (the region populated by low-SR spontaneous rate fibers), a reduction in the neural gain was observed especially for the largest corresponding to higher levels of sound stimulation (Fig 4H). Worthy of note is the lack of significant change in the CAP amplitude-intensity functions at all narrow-band frequency ranges tested (Fig 5A–5D).

### 3. Quantification of ribbon-synapses per inner hair cell

**Fig 6:**
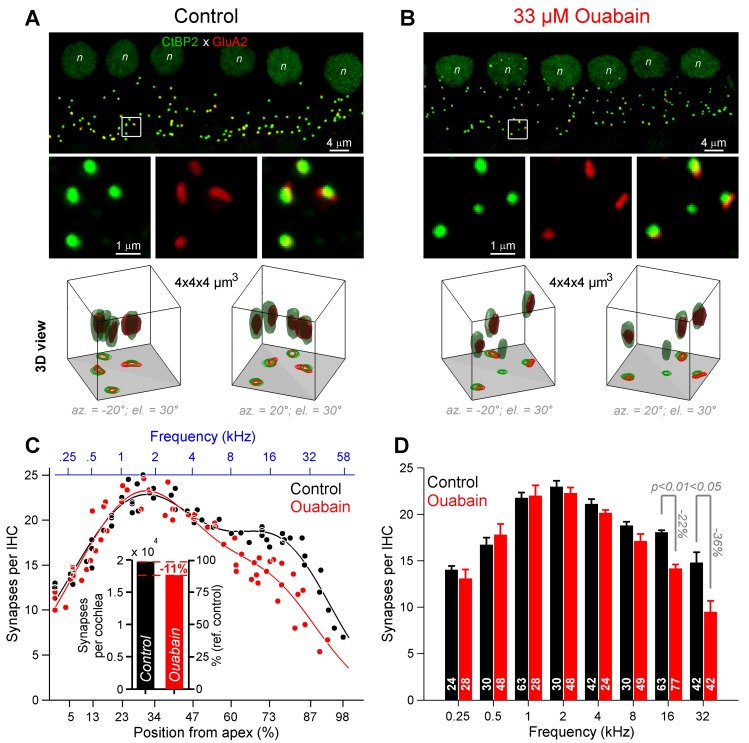
Synapse counts in control and ouabain-poisoned cochleae. **(A, B)** Confocal microscopy of immunolabeled CtBP2 (green) and GluA2 (red) from the 16-kHz encoding region in artificial perilymph control (**A**) and ouabain-poisoned cochleae (**B**). *Top panel*: enlarged view of inner-hair-cell innervation (6 IHCs; n indicates the nucleus of IHCs). *Middle panel*: *z*-projection of the white square shown above (4 μm × 4 μm), showing CtBP2 and GluA2 immunolabeling alone or together (merged). *Bottom panel*: Three-dimensional (3D) views of the white square shown above (4 μm × 4 μm × 4 μm). Note the presence of an orphan ribbons in ouabain-poisoned condition (**B**, 12 ± 2% in the basal end (>5.6 kHz) against 1 ± 0.6% in the apical end (<5.6 kHz). (**C**) Number of synapses per IHC along the gerbil tonotopic axis [24] in control and ouabain-poisoned cochleae (black, control, 5 cochleae, 324 IHCs, 5790 synapses; red, ouabain, 5 cochleae; 344 IHCs, 5494 synapses). Each dot represents the average over 6 consecutive IHCs [14]. Black and red curves are fits using the sum of two Gaussian models (control, black, *f(x)* = 22.6×exp(-((*x*-30)/35.2)^2^) + 14.2×exp(-((*x*-78.6)/24.1)^2^), *r*^2^ = 0.92; ouabain, red, *f(x)* = 22.9×exp(-((*x*-30)/33.4)^2^) + 9.7×exp(-((*x*-75.2)/23.6)^2^), *r*^2^ = 0.88, with *x* the position from the apex in percent). *Inset*: Estimates of the number of synapses per cochlea calculated from IHC and synapse counts. (black, control: 19,659 synapses/cochlea; red, ouabain: 17,568 synapses/cochlea). (**D**) Number of synapses per IHC pooled per octave band, in control (black) and ouabain-poisoned (red) cochleae. Numerical values indicate the number of IHCs for which the number of synapses was assessed. Data were expressed as the mean ± SEM, *P*<0.05, *P*<0.01, two-way ANOVA test followed by *post hoc* Tukey’s test.

The high-frequency reduction of neural gain index in ouabain-treated gerbils was then demonstrated to correspond to a reduction in the number of synapses beyond the 5.6 kHz cochlear region. Using immunohistochemistry, the ribbon-anchored synapse number was approximated along the tonotopic axis by the juxtaposition of the IHCs presynaptic ribbon organelle and the postsynaptic density (Fig 6A and 6B). In artificial prerilymph control cochleae (*n* = 5), the distribution of synapses per IHC was bimodal, with a prominent number of synapses in the 2 and the 16 kHz cochlear regions (Fig 6C), which is consistent with the bimodal shape of the iso-level neural gain at 60 dB SPL (Fig 4A). Six days after infusion of 33 μM ouabain into the round window niche (*n* = 5), the distribution of synapses per IHC along the tonotopic axis displayed a single mode centered on 2 kHz (Fig 6C and 6D). Interestingly, the total number of synapses decreased by 11% in ouabain-poisoned cochleae (Fig 6C inset), with the greatest reduction in the 16 and 32 kHz regions (-22% and -36%, respectively, Fig 6D). This is consistent with the distribution of low-SR fibers in the base of the gerbil cochlea [2,14].

## Discussion

The present study demonstrated that monitoring the mass potentials recorded at the round window niche makes possible the detection of low-SR fibers while the monitoring of CAP amplitude fails. In contrast to other mammals, in which the SR-based distribution of ANFs along the tonotopic axis is relatively homogenous [37], the gerbil cochlea displays considerable variations with a prevalence of high-SR (SR > 18 spikes/s) below 3.5 kHz and a more balanced distribution of high-, medium- (0.5 < SR < 18 spikes/s) and low-SR (SR < 0.5 spike/s) fibers above 3.5 kHz [2,14,22,23,24]. The iso-level frequency response of the neural index at 60 dB SPL displays a bimodal shape peaking at 2 kHz and 16 kHz, as a probe-stimulation level of 60 dB SPL suffices to activate all the ANFs independently of their SR [2] with a weak spread of excitation [38]. Consistently, when the level of stimulation decreases below the threshold of low-SR fibers (threshold of 30–40 dB SPL, [2]), the iso-level frequency response curve becomes unimodal with a peak at 2 kHz reflecting mainly the activation of high-SR fibers (threshold of 10–20 dB SPL, [2]). This hypothesis is confirmed after the ablation of low-SR fibers, in which the iso-level neural gain index remains unimodal even at 60 dB SPL. Taken together, the neural gain index at 2 and 16 kHz are the signatures of differing compositions of ANF pools at the base and the apex of the gerbil’s cochlea, i.e., the shape of the iso-level response of neural gain index represents the cumulative activation of ANFs according to their SR and threshold.

### Different contributions in the summed response to alternating stimulus polarities

Mass potential recorded at the round window contains both a neural arising from ANFs, and a microphonic component originating from transduction currents of hair cells [21]. Using appropriate sound stimulation and signal processing protocols, we are able to eliminate the microphonic component generated by hair cells. This is of particular interest below 4 kHz, where cochlear microphonic would overlap with the “900 Hz-peak” and thus interfering with the measurement of the neural activity. Unfortunately, this procedure also reduces the spectral component in low-frequency region (< 2 kHz) where fibers are phase-locked with fine structure of the stimulus (1/3 octave band noise in this study). Indeed, neural responses arising from the phase-locked activity of ANFs, called neurophonic response can be subdivided in two components: the neurophonic related to the stimulus fine-structure and the neurophonic related to the stimulus envelope. In our study, both types of neurophonic contribute to the mass potential response recorded at the round window, since we used a small band of noise rather than a pure tone. At low frequency (below 4 kHz in gerbil, [39]), the neurophonic is related to the fine-structure, and at high frequency (above 4 kHz) it is only related to envelope (see [10] for a review). At low frequency, the second stimulation within each pair of stimuli occurs in opposite phase with the first and the ANFs respond in phase locking to fine structure, but with a shift of ½ cycle of stimulation. The summation of opposite phase responses leads to the reduction of the first harmonic neural activity, whereas the second harmonic at twice the frequency of the probe was maintained [16,17,34,35]. Consequently, for frequencies below 2 kHz, the gain of neural activity is tricked because of the phase locking to fine structure. Therefore, second harmonic [34,35] or forward masking procedures [16,17] would be more appropriated to probe low-CF fibers.

### Contribution of the low-SR fibers to the mass potentials recorded at the round window

Whereas low-SR fibers are not detectable in the CAP of the auditory nerve, we report a reduction of the neural gain in the frequency range where the low-SR fibers are located, mostly in the basal turn [2,22,23,24]. Our current hypothesis is because CAP only captures the first spike of the fibers responding in synchrony to the acoustic stimulation, while the neural gain measured herein relies on steady state response. In our experiments, neural activity was measured over the last hundreds of milliseconds of the acoustic stimulation. In contrast, the first spikes of ANFs firing in synchrony with the onset-stimulus yield the sound-evoked CAP. In gerbils, the degree of synchronization of ANFs (i.e. first spike latency jitter) is positively correlated with SR [14]. Consequently, the low-SR fibers do not contribute to the CAP, and their loss is thus not detected in the CAP. Accordingly, 33 μM ouabain led to a steady-state neural activity reduction in the high frequency region of the cochlea where the low-SR fibers are located, while the CAP did not change.

## Conclusion

Altogether, our data support the idea that the use of electrophysiological methods based on first spike response is not suitable to track the loss of low-SR fibers. This result questions the use of CAP and the first positive wave (P1) of the auditory brainstem responses in experimental and clinical assessments, as both metrics only reflect the first spike of an assembly of fibers which fired in synchrony. Here, we propose that mass potentials at round-window, which do not rely on the onset response of fibers but rather the steady state discharge rate, constitute a more promising avenue to probe the SR-based distribution of ANFs in humans using transtympanic electrocochleography [40], and in other species in which direct single-unit recordings are difficult to achieve or are not feasible.
